# Maintenance treatment with the immunomodulator MGN1703, a Toll-like receptor 9 (TLR9) agonist, in patients with metastatic colorectal carcinoma and disease control after chemotherapy: a randomised, double-blind, placebo-controlled trial

**DOI:** 10.1007/s00432-014-1682-7

**Published:** 2014-05-10

**Authors:** Hans-Joachim Schmoll, Burghardt Wittig, Dirk Arnold, Jorge Riera-Knorrenschild, Dieter Nitsche, Hendrik Kroening, Frank Mayer, Johannes Andel, Reinhard Ziebermayr, Werner Scheithauer

**Affiliations:** 1 Department of Internal Medicine IV, Oncology/Hematology, University Clinic Halle (Saale), Center for Cell and Gene Therapy, Martin Luther University Halle-Wittenberg, Ernst-Grube-Str. 40, 06120 Halle, Germany; 2 Foundation Institute Molecular Biology and Bioinformatics, Freie Universitaet Berlin, Arnimallee 22, 14195 Berlin, Germany; 3 Department of Medical Oncology and Hematology, Tumor Biology Center of the Albert-Ludwigs-University, Breisacher Str. 117, 79106 Freiburg, Germany; 4 Hematology, Oncology and Immunology, University Hospital of Giessen and Marburg, Baldingerstraβse 1, 35043 Marburg, Germany; 5 Hematology, Oncology, Gastroenterology, Barmherziger Schwestern Linz, Seilerstätte 4, 4010 Linz, Austria; 6 Hämatologie und Onkologie, Schwerpunktpraxis für Hämatologie und Onkologie, Hasselbachplatz 2, 39104 Magdeburg, Germany; 7 Department of Internal Medicine II, University of Tuebingen Medical Center, Ottfried-Mueller-Str. 10, 72076 Tuebingen, Germany; 8 Internal Medicine II, Hospital of Steyr, Sierninger Str. 170, Steyr, Austria; 9 Interne Hämatologie mit Stammzeltransplantation und medizinischer Onkologie, Elisabethinen Linz, Fadingerstraβe 1, 4020 Linz, Austria; 10 Department of Internal Medicine I and Comprehensive Cancer Center, Medical University of Vienna, Waehringer Güertel 18-20, 1090 Vienna, Austria

**Keywords:** MGN1703, Immunomodulator, TLR9 agonist, Colorectal cancer, Maintenance

## Abstract

**Purpose:**

This phase II study evaluated the synthetic DNA-based immunomodulator and Toll-like receptor 9 agonist MGN1703 as maintenance treatment in metastatic colorectal carcinoma (mCRC).

**Methods:**

Fifty-nine patients with mCRC and disease control after standard first-line chemotherapy were randomised to MGN1703 60 mg (*N* = 43) or placebo (*N* = 16).

**Results:**

The hazard ratio (HR) for the primary endpoint [progression-free survival (PFS) from the start of maintenance] was 0.56 (95 % CI 0.29–1.08; *P* = 0.07) and 0.55 (95 % CI 0.3–1.0; *P* = 0.04) by independent and investigator review, respectively. MGN1703 significantly improved PFS measured from the start of induction therapy versus placebo on independent (HR 0.49; 95 % CI 0.26–0.94; *P* = 0.03) and investigator review (HR 0.50; 95 % CI 0.31–1.02; *P* = 0.02). Overall survival (OS) data remain immature (HR 95 %; 95 % CI 0.3–1.5; *P* = 0.29) with 28/43 patients alive after a medium follow-up of >17 months. Retrospective subgroup analysis showed a significant effect of MGN1703 on PFS versus placebo in patients with greater than median tumour size reduction and normalised carcinoembryonic antigen concentrations following induction therapy, and in patients with elevated activated NKT cells ≥3.08 %. Adverse events were mild to moderate and limited to injection-site reactions or linked to general immune system activation.

**Conclusions:**

MGN1703 maintenance treatment was well tolerated and appears to induce durable and prolonged PFS and disease control in a subgroup of patients with mCRC following induction therapy. Activated NKT cells may be a predictive biomarker for selecting patients likely to benefit more from MGN1703.

**Electronic supplementary material:**

The online version of this article (doi:10.1007/s00432-014-1682-7) contains supplementary material, which is available to authorized users.

## Introduction

**Fig. 1:**
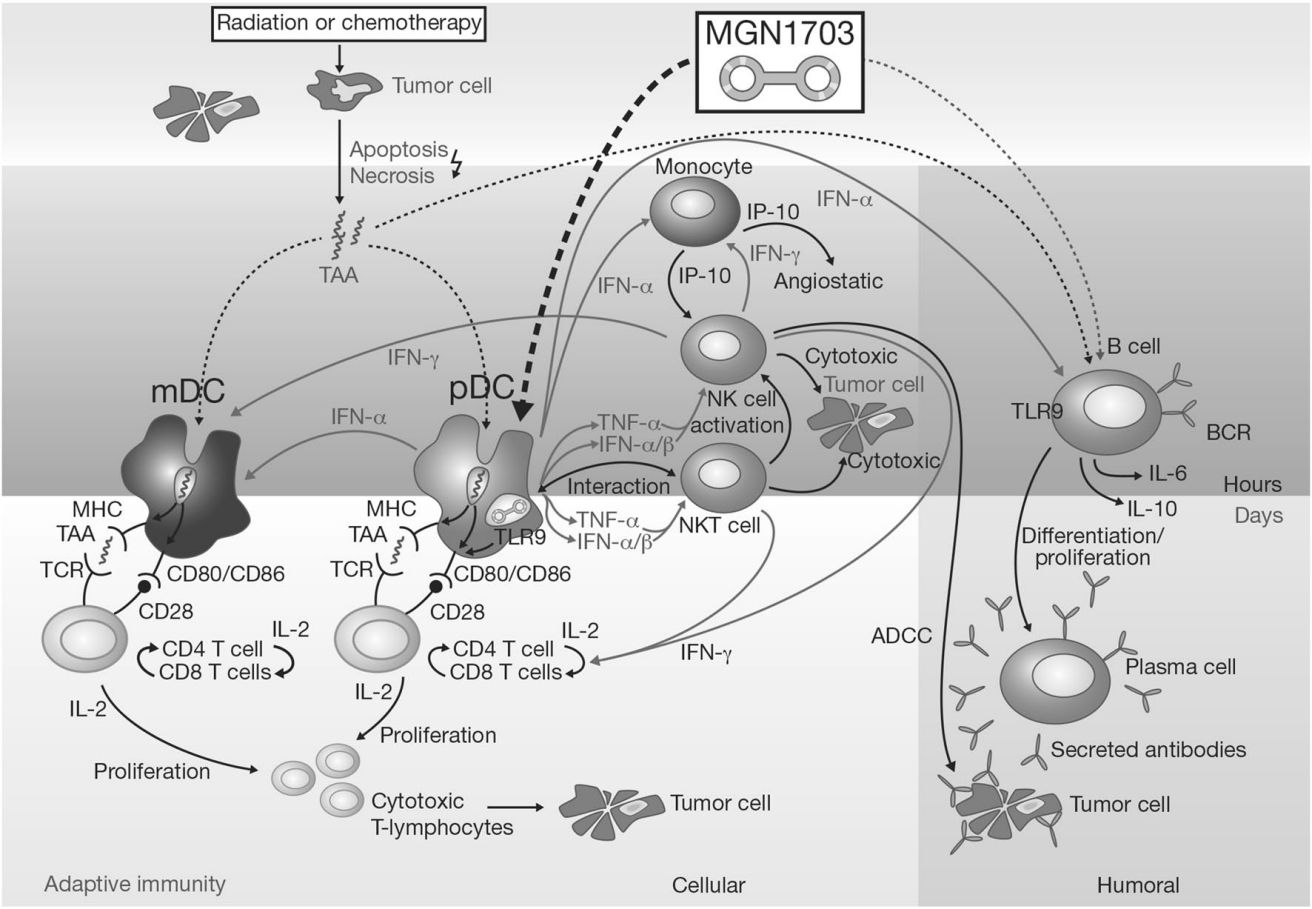
Mechanism of action of MGN1703. *ADCC* antibody-dependent cell-mediated cytotoxicity, *BCR* B-cell receptor, *IFN* interferon, *IL* interleukin, *mDC* myeloid dendritic cells, *MHC* major histocompatibility complex, *NK cell* natural killer cell, *NKT cell* natural killer T cell, *pDC* plasmacytoid dendritic cells, *TAA* tumour-associated antigens, *TCR* T-cell receptor, *TLR9* Toll-like receptor 9

In recent years, a number of agents have been developed with the aim of harnessing the inherent anti-cancer capabilities of the immune system. Most efforts have focused on the activation of adaptive immunity, as seen with ipilimumab—a cytotoxic T lymphocyte-associated antigen 4 inhibitor active in melanoma—and with other checkpoint inhibitors that target the programmed cell death 1 pathway (Hodi et al. 2010; Robert et al. 2011; Topalian et al. 2012; Tournigand et al. 2012; Brahmer et al. 2012). An additional immunomodulatory approach involves stimulation of innate immunity. MGN1703 is a novel, synthetic, dumb-bell-shaped, covalently closed DNA molecule (Weihrauch et al., submitted), which activates the innate immune system via Toll-like receptor 9 (TLR9) (Fig. 1). The TLR9 receptor is expressed on plasmacytoid dendritic cells (pDC) and B cells and recognises non-methylated cytosine–guanine dinucleotide (CG) motifs in bacterial, viral and mitochondrial DNA (Schmidt et al. 2006a; Krieg 2002; Kanzler et al. 2007; Ahmad-Nejad et al. 2002). TLR agonists can stimulate innate antitumour mechanisms, including activation of natural killer T (NKT) cells, monocytes and macrophages, and induction of cytokines (Kanzler et al. 2007; Carpentier et al. 2006; Pashenkov et al. 2006; Friedberg et al. 2005). TLR agonists containing non-methylated CG motifs have exhibited limited, transient adverse effects and some efficacy in clinical trials in various tumour types (Kanzler et al. 2007; Carpentier et al. 2006; Pashenkov et al. 2006; Friedberg et al. 2005).

MGN1703 has been investigated as adjuvant therapy in two trials (Weihrauch et al. 2005; Wittig et al. 2001). In the first, MGN1703 was used in combination with chemoimmunotherapy in patients with metastatic colorectal carcinoma (CRC) (Weihrauch et al. 2005); in the second, patients with metastatic solid tumours of colon, renal cell, or melanoma origin received autologous gene-modified cells with adjuvant MGN1703 (Wittig et al. 2001). In both cases, regimens were well tolerated with mild, transient side effects, although MGN1703 doses were low (Weihrauch et al. 2005; Wittig et al. 2001). More recently, a phase I trial showed that twice-weekly subcutaneous applications of single-agent MGN1703 (up to 60 mg) were well tolerated in 28 patients with metastatic solid tumours (Weihrauch et al., submitted). Six patients had stable disease (prolonged in three cases), and one with refractory CRC had a partial response.

The current study evaluated MGN1703 as maintenance treatment in patients with metastatic CRC who had disease control after induction chemotherapy. Failure of a different TLR9 agonist to improve outcomes in advanced non-small cell lung cancer when added to chemotherapy suggests that the supportive immune response initiated by TLR9 agonists may firstly require release of tumour-associated antigens (Hirsh et al. 2011). A decreased tumour burden together with a chemotherapy-free interval allowing recovery of immune cells may also facilitate a more effective immune response (Hirsh et al. 2011). This type of immunotherapy may therefore prove successful when given sequentially after chemotherapy. Treatment for metastatic CRC has improved significantly over the last two decades (Chu 2012), and the potential value of maintenance therapy is being extensively investigated (Strickler and Hurwitz 2012; Díaz-Rubio et al. 2012; Tournigand et al. 2012; Koeberle et al. 2013; Koopman et al. 2014).

IMPACT, a randomised, placebo-controlled, double-blind, phase II study, evaluated the efficacy and safety of subcutaneous MGN1703 (60 mg twice-weekly) as maintenance therapy following successful first-line induction therapy in metastatic CRC. This was the first placebo-controlled trial to prospectively investigate the impact of an immunomodulator as maintenance therapy in metastatic CRC and was based on the hypothesis that patients with disease control could benefit from immunotherapy.

## Methods

### Study population

The study recruited males or females aged over 18 years with histologically confirmed CRC that had been radiologically confirmed before starting first-line therapy as unresectable and advanced disease (American Joint Committee on Cancer stage IV). To be eligible, patients were also required to have previous first-line therapy with fluoropyrimidine plus irinotecan or oxaliplatin, with or without bevacizumab, for 4.5–6 months (treatment with oxaliplatin or irinotecan for at least 3 months); disease control after first-line therapy, defined as objective response or stable disease; at least one measurable lesion according to revised Response Evaluation Criteria in Solid Tumors (RECIST) (Eisenhauer et al. 2009); Eastern Cooperative Oncology Group (ECOG) performance status 0 or 1; and adequate bone marrow, liver and kidney function. Patients were excluded if they had tumour progression after first-line therapy; more than one previous line of systemic chemotherapy for metastatic CRC; known central nervous system metastases; history of autoimmune disease or immune deficiency; active or uncontrolled infections; transfusion-dependent anaemia; concurrent chronic systemic immune therapy or immunosuppressant medication including steroid treatment; chemotherapy or immunotherapy within the 2 weeks before randomisation or radiotherapy 6 months before randomisation. All patients gave written, informed consent.

### Study design and treatments

This randomised trial took place at 22 centres in Austria, France, Germany and Russia (ClinicalTrials.gov identifier: NCT01208194). The primary endpoint was progression-free survival (PFS), measured from the date of randomisation to progression on maintenance therapy. Secondary endpoints included PFS measured from the start of induction therapy to progression on maintenance; overall survival (OS) from randomisation and start of induction therapy; objective response rate per RECIST during maintenance treatment; safety; and biomarkers for efficacy, including immunological response. Patients were randomised in a 2:1 ratio to MGN1703 60 mg or placebo, both given subcutaneously twice-weekly until disease progression, unacceptable toxicity, appearance of exclusion criteria, withdrawal of patient consent, or death. The study was approved by the relevant Investigational Review Boards or Ethics Committees and was run in accordance with the Declaration of Helsinki and Good Clinical Practice Guidelines.

### Randomisation and masking

A randomisation list was generated using a standard computer programme and was designed in blocks of six patients. Treatment was allocated centrally; the sponsor provided sites with a patient number, which uniquely identified the treatment to be administered. The study was double-blind, with one copy of the complete randomisation code held by Mologen AG until study end and a second provided to a Data Safety Monitoring Board (DSMB). Investigators received a sealed random code envelope for each individual patient number, which was opened only in an emergency.

### Assessments

Tumour response was assessed by computed tomography scan using the revised RECIST (Eisenhauer et al. 2009) at baseline, at weeks 12, 18 and 24, and every 12 weeks thereafter. Assessment of scans by local investigators was confirmed by review by two independent radiologists. Regular safety assessments included physical examination, haematology and clinical chemistry tests, vital signs, electrocardiogram and ECOG performance status. Adverse events were monitored regularly and graded using the National Cancer Institute Common Toxicity Criteria for Adverse Events version 4.0.

### Immunotherapy biomarker study

Whole blood samples were collected for the analysis of immunotherapy biomarkers at baseline and after 3, 6, 12 and 24 weeks, or at the end of study visit if after 24 weeks. Blood samples were shipped to the central laboratory (Labor Berlin GmbH, Germany) at room temperature and were analysed by fluorescence activated cell sorting (FACS) within 24 h. Frequency and activation status of the following immune cells were assessed by FACS: monocytes, B lymphocytes, T lymphocytes, NKT cells, NK cells, plasmacytoid dendritic cells and myeloid dendritic cells.

### Data analyses

Sample size was estimated using Nquery version 7.0; the calculation assumed that PFS would increase from 3 months with placebo to 6 months with MGN1703, that at 6 months, the hazard ratio (HR) for progression would be 0.5 and an alpha level of 0.05 with 80 % power. An estimated 129 patients were required (86 and 43 patients randomised to MGN1703 and placebo, respectively).

The primary analysis of efficacy endpoints was on an intent-to-treat basis. Efficacy was also analysed in a subgroup based on the per-protocol population but which excluded patients with abnormal prognostic laboratory parameters at baseline and/or the first visit on study. Safety analyses used the as-treated population (all patients who received at least one dose of study medication, with treatment assignments designated according to the actual study treatment received). The DSMB monitored safety data and advised on whether the trial could be continued. One interim analysis was planned after 60 patients had been treated for 4.5 months. PFS and OS were estimated using Kaplan–Meier methods and medians for each variable presented with 95 % confidence intervals (CIs). PFS and OS were compared between treatment groups using the log-rank test. The proportion of patients in each group who achieved an objective tumour response, defined as the sum of complete and partial responses, was calculated.

To identify patient subgroups most likely to obtain benefit from MGN1703 with respect to PFS, pre-planned analyses of immunotherapy biomarkers and patient characteristics at baseline were performed. Cox regression analysis and receiver operating characteristic analysis were used to identify biomarkers and optimal cut-off levels appropriate for the comparison of PFS. Methodology of the baseline characteristics analysis is described in the Appendix.

### Role of the funding source

The study sponsor participated in study design and data collection and interpretation. This report was written by the corresponding author with contribution and review by all co-authors, including those employed by the sponsor. The corresponding author had full access to all study data and had final responsibility for the decision to submit the report for publication.

## Results

### Patients, demographics and baseline characteristics

**Fig. 2:**
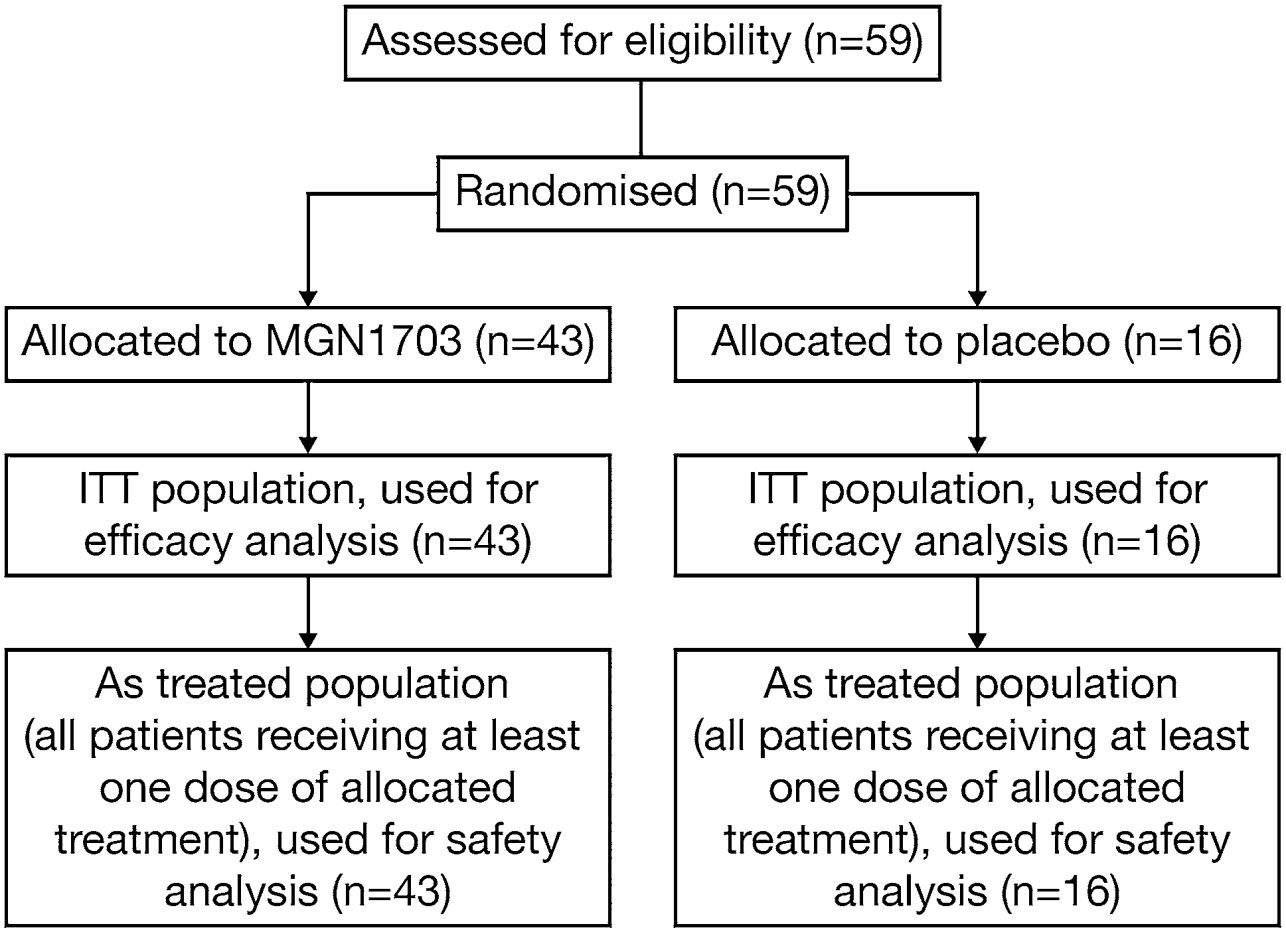
Trial profile. *ITT* intent to treat

Between June 2010 and May 2012, 59 patients were randomised to receive MGN1703 (*N* = 43) or placebo (*N* = 16) (Fig. 2). Patient baseline demographics and characteristics were generally well balanced between treatment groups (Table 1).

**Table 1:** Patient demographic and disease characteristic at baseline (intent-to-treat cohort)

Characteristic	MGN1703	Placebo
	(*N* = 43)	(*N* = 16)
Age, median (range)	65.0 (39–80)	67.5 (44–80)
Gender: male/female, *n* (%)	21/22 (49/51)	8/8 (50/50)
ECOG performance status: 0/1, *n* (%)	29/14 (67/33)	10/6 (63/37)
Site of primary tumour, *n* (%)		
Colon	24 (56)	9 (56)
Rectum	13 (30)	6 (17)
Both	6 (14)	1 (6)
Site of metastatic disease^a^, *n* (%)		
Liver only	15 (35)	6 (38)
Lung only	4 (9)	1 (6)
Other	24 (56)	9 (56)
Surgery: primary in situ	12 (28)	5 (31)
Median duration (range) of induction therapy, months	5.4 (3–10)	5.3 (4–7)
Prior induction regimen, *n* (%)		
FOLFOX/XELOX + bevacizumab	16 (37)	7 (44)
FOLFIRI/XELIRI + bevacizumab	21 (49)	8 (50)
FOLFOX/XELOX alone	6 (14)	1 (6)
Best response to induction therapy, *n* (%)		
CR/PR	29 (67)^b^	14 (88)
SD	13 (30)	2 (12)

*CR* complete response, *ECOG* Eastern Cooperative Oncology Group, *PR* partial response, *SD* stable disease

^a^Assessed before induction therapy

^b^Difference to 100 %: missing values

Following a planned interim analysis, recruitment was halted in May 2012 before reaching the recruitment target of 129 patients. The primary reason for stopping the trial was the slow recruitment, which could not be accelerated, despite several attempts by the sponsor.

At the time of this analysis, median duration of follow-up for the MGN1703 group was 17.7 months (95 % CI 13.8–19.6) and for placebo was 16.5 months (95 % CI 15.3–21.6). Median treatment duration was 3.2 months (range 1–26) and 2.9 months (range 1–12), respectively. All patients had discontinued; the most common reason for discontinuation was disease progression, reported in 31 (72 %) and 14 (88 %) patients in the MGN1703 and placebo groups, respectively. Eight patients discontinued study for clinical signs of relapse (such as fever, increasing biomarkers and local obstruction) that could not be confirmed by the independent review. Four patients continued to receive treatment with MGN1703 following the study.

### Efficacy and response

**Fig. 3:**
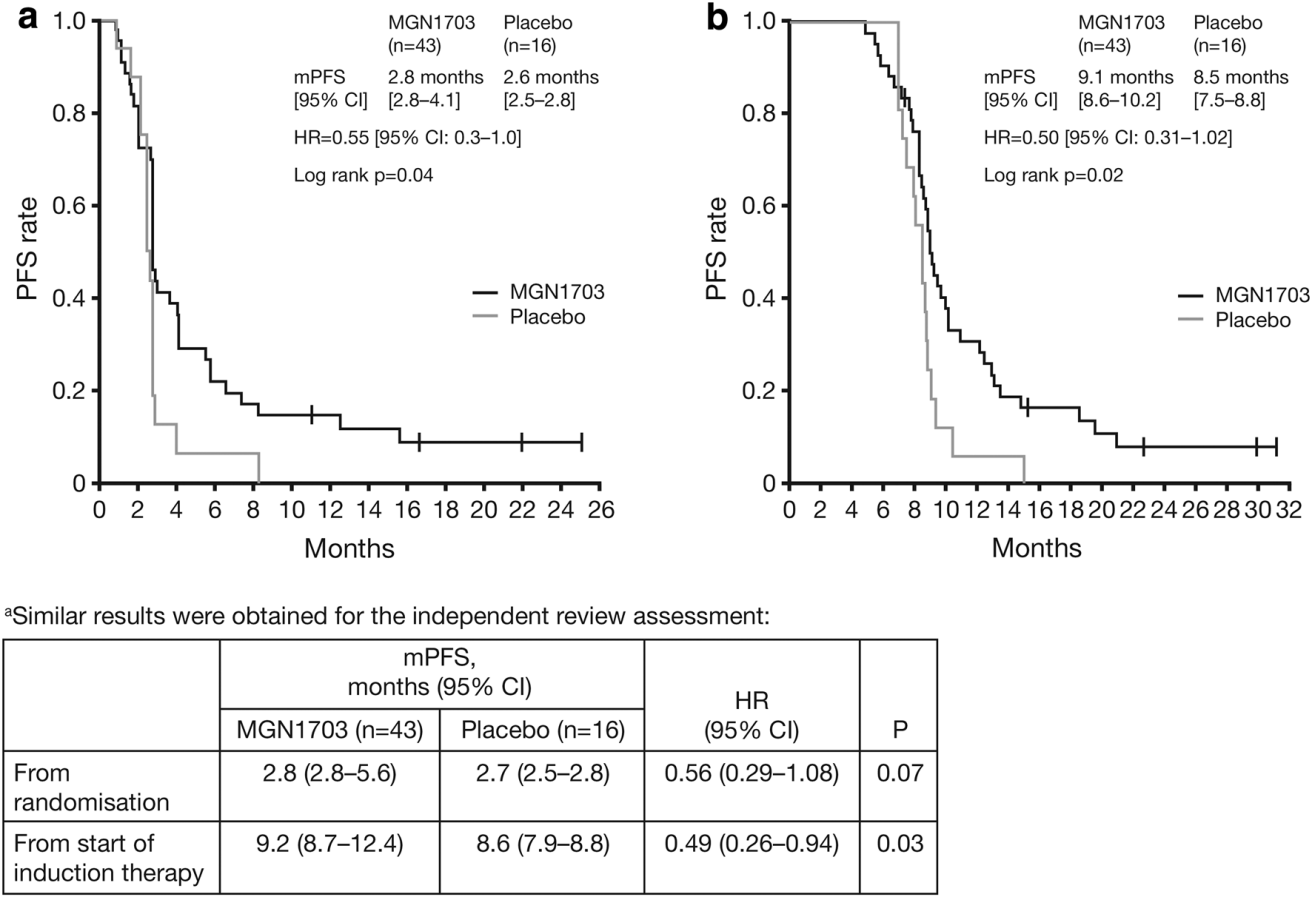
PFS by treatment group from **a** randomisation and **b** start of induction therapy (investigator assessment^a^). *CI* confidence interval, *HR* hazard ratio, *mPFS* median progression-free survival, *PFS* progression-free survival

Hazard ratio (HR) for investigator assessment of the primary endpoint was 0.55 (95 % CI 0.3–1.0; *P* = 0.04), with MGN1703 maintenance treatment associated with durable PFS in some patients (Fig. 3a); median PFS was 2.8 months (95 % CI 2.8–4.1) with MGN1703 and 2.6 months (95 % CI 2.5–2.8) with placebo. HR for an independent review assessment of the primary endpoint was 0.56 (95 % CI 0.29–1.08; *P* = 0.07) (see Fig. 3 footnote).

When measured from the start of induction therapy, PFS was significantly improved with MGN1703 versus placebo both with independent and investigator assessment (HR 0.49; 95 % CI 0.26–0.94; *P* = 0.03 and HR 0.50; 95 % CI 0.31–1.02; *P* = 0.02; Fig. 3b and footnote). HR for PFS from randomisation in the protocol-defined subgroup population with ‘good risk’ characteristics was 0.52 (95 % CI 0.26–1.04; *P* = 0.0526), corresponding to a median PFS of 3.0 months (95 % CI 2.8–6.6) with MGN1703 (*N* = 38) and 2.8 months (95 % CI 2.5–2.8) with placebo (*N* = 15).

Overall survival (OS) data were still immature after a median follow-up of over 17 months (Appendix Fig. S1a), with more than 50 % of patients censored. Measured from randomisation, median OS was 22.6 months (95 % CI 14.9–not reached [NR]) with MGN1703 and 15.1 months (95 % CI 10.6–NR) with placebo (HR 0.63; 95 % CI 0.3–1.5; *P* = 0.2886; Appendix Fig. S1a). Median OS from the start of induction therapy was 26.3 months (95 % CI 21.0–NR) and 21.2 months (95 % CI 16.5–NR) for MGN1703 and placebo, respectively (HR 0.65; 95 % CI 0.3–1.6; *P* = 0.3339; Appendix Fig. S1b).

Three patients in the MGN1703 group had a confirmed objective tumour response (objective response rate 7.0 %) during maintenance treatment, observed 3, 9 and 9 months after randomisation. At the time of this analysis, all three patients, plus a fourth patient in complete remission after induction chemotherapy, remain stable without relapse, having been on treatment for 16–30 months. One placebo-treated patient had a response (objective response rate 6.3 %) after 3 months (response duration, 6 months).

### Analyses of immunological biomarkers

**Fig. 4:**
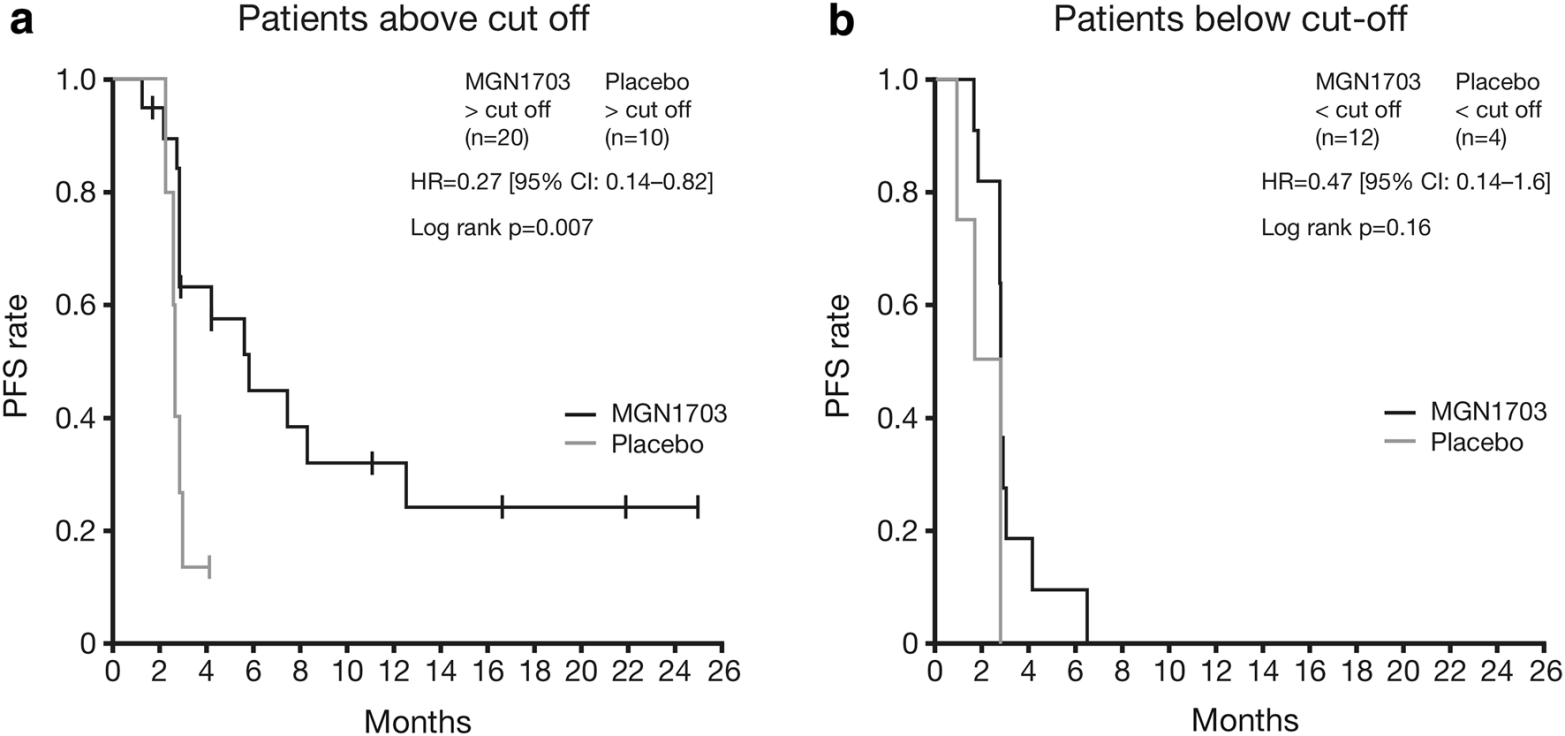
PFS from randomisation by treatment group for patients with NKT cell counts ≥3.08 % (**a**) or <3.08 % (**b**). *CI* confidence interval, *HR* hazard ratio, *NKT* natural killer T, *PFS* progression-free survival

Forty-six patients were included in a pre-planned analysis of potential immunological biomarkers (32 and 14 patients from the MGN1703 and placebo groups, respectively). In patients with high (≥3.08 %) activated NKT cell counts at baseline, MGN1703 was associated with significantly greater PFS than placebo (HR 0.27; 95 % CI 0.14–0.82; *P* = 0.007; Fig. 4a). This phenomenon was not observed in patients with activated NKT cell counts below this cut-off (HR 0.47; 95 % CI 0.14–1.6; *P* = 0.16; Fig. 4b).

Evidence for the activation of the immune system in patients treated with MGN1703 was provided by observed increases in the proportion of activated monocytes and pDC in these patients (Appendix).

### Analyses of baseline patient characteristics predictive for progression-free survival

In the analyses of baseline patient characteristics, two of three factors included in the Cox proportional hazards model had statistically significant effects on PFS: reduction in tumour size at the end of induction therapy (>median versus ≤ median; HR 1.015 [95 % CI 1.002–1.029; *P* = 0.0238]) and carcinoembryonic antigen (CEA) concentration at the end of induction therapy (<upper limit of normal [ULN] versus ≥ ULN; HR 0.326 [95 % CI 0.159–0.668; *P* = 0.0022]). Resection of primary tumour (yes versus no) was not significant (HR 0.851; 95 % CI 0.385–1.884; *P* = 0.6921).

In patients with a reduction in tumour size following induction therapy greater than the median reduction, PFS was significantly longer with MGN1703 than placebo but this effect of MGN1703 was not observed in patients with a reduction in tumour size less than median (Appendix Fig. S2a). Similarly, in patients with CEA concentrations within the normal range, PFS was significantly longer with MGN1703 than placebo but no such effect was observed in patients with CEA above the normal range (Appendix Fig. S2b). PFS was also significantly extended with MGN1703 in patients with response after induction but not in patients with stable disease (Appendix Fig. S2c). These data should be treated with some caution, however, since patient numbers are small, particularly in the placebo group.

### Safety and tolerability

In the MGN1703 and placebo groups, respectively, 79 and 38 % of patients experienced any treatment-emergent adverse event; overall incidence of grade 3/4 adverse events was 26 and 13 %, respectively. Treatment-related adverse events were reported in 33 and 12.5 % of MGN1703- and placebo-treated patients, respectively, while grade 3/4 adverse events regarded as treatment-related were rare (2 and 8 %, respectively; *n* = 1 in each group). One patient discontinued MGN1703 because of an adverse event (sensory neuropathy; no discontinuations in the placebo group).

Grade 3/4 treatment-emergent adverse events observed in the MGN1703 group included ileus (*n* = 4), hypertension (*n* = 2), worsening of hypertension (*n* = 2), neutropenia, aspartate aminotransferase increase, sensory polyneuropathy and sepsis (each *n* = 1). The case of polyneuropathy was considered possibly related to MGN1703, but was more likely to have been associated with previous oxaliplatin chemotherapy. Among five patients with serious adverse events, two had ileus, a third had ileus with sepsis and another had ileus with alimentary toxicosis; none of these events were considered related to treatment. The fifth patient was admitted to hospital with atypical pneumonia (grade 2), which was considered possibly related to MGN1703 treatment. In the placebo group, grade 3 adverse events comprised papular exanthema, food poisoning and coxarthrosis (each *n* = 1); there were no grade 4 adverse events and none were considered serious.

Treatment-related adverse events of interest because of the mode of action of MGN1703 are summarised in Table 2. These were typically mild to moderate (grade 1 or 2) and in the MGN1703 group included most commonly flu-like symptoms and injection-site reactions. Mild local reactions (pain, redness, itching or induration) occurred in 77 and 6 % of patients in the MGN1703 and placebo groups, respectively. These symptoms were limited at the site of injection and did not result in treatment interruption.

**Table 2:** Summary of treatment-emergent adverse events of particular interest due to MGN1703 mechanism of action

AE, number of patients (%)	MGN1703 (*N* = 43)		Placebo (*N* = 13)	
	Grade 1/2	Grade 3/4	Grade 1/2	Grade 3/4
Flu-like symptoms	6 (13.9)	–	1 (7.7)	–
General pain	4 (9.3)	–	1 (7.7)	–
General rash, itching, paraesthesia	4 (9.3)	–	–	1 (7.7)
Injection-site reaction	2 (4.6)	–	1 (7.7)	–
Atypical pneumonia	2 (4.6)	–	–	–
Polyneuropathy	1 (2.3)	1 (2.3)	–	–
ANA increased	1 (2.3)	–	1 (7.7)	–
Fatigue	1 (2.3)	–	–	–
Hypertension	1 (2.3)	–	–	–

*AE* adverse event, *ANA* antinuclear antibody

## Discussion

The IMPACT trial showed that MGN1703 maintenance treatment was well tolerated and appears to induce durable PFS and disease control in selected patients with advanced CRC following induction therapy.

Findings from the study reflect the complications and challenges faced in assessing clinical response to immunotherapies. In the primary endpoint analysis, Kaplan–Meier curves separated after the median point; similar findings have been reported in other recent studies in metastatic CRC, for example, in populations with mixed *KRAS* mutation status treated with epidermal growth factor receptor inhibitors (Saif and Shah 2009; Ramos et al. 2008). Therefore, the activity of MGN1703 may be limited to a specific subpopulation of patients who achieve a prolonged response to treatment. Indeed, results from the secondary analyses indicate that three possible subgroups existed. Firstly, the greatest benefit with MGN1703 was observed in patients who achieved shrinkage of their tumour after induction chemotherapy. This is consistent with the theory that immunomodulatory treatment is more likely to be successful in patients with relatively low tumour burden. Similarly, normal CEA values after induction therapy also had predictive value. Finally, results from the immunological biomarker analysis reported a significant effect of MGN1703 on PFS relative to placebo in patients with levels of activated NKT cells ≥3.08 % but not in those with levels below this cut-off. Activated NKT cells may be a potential biomarker for selecting patients more likely to benefit from MGN1703 treatment.

Two assessments of response were used in the trial (investigator and independent review). Primary endpoint results with the two assessment methods were similar, although *P*-values decreased with the investigator assessment relative to independent review (0.04 versus 0.07), reflecting a decrease in statistical uncertainty caused by a reduction in the number of censored patients. In the independent review, 13 patients were censored (eight local relapses as assessed by investigators could not be confirmed on scans by the independent review). The investigator assessment included these eight patients so that only five patients were censored. Such inaccurate identification of relapse is indicative of the challenges involved in measuring responses to immunotherapy. Immunomodulators are associated with unique patterns of response versus conventional therapies. In studies of ipilimumab, for example, disease regression was sometimes not seen for several months and, in other cases, lesion growth was observed due to immune-related inflammation not disease progression (Weber et al. 2012).

Immune-related response criteria have now been defined (Wolchok et al. 2009), although traditional RECIST criteria, which may not detect all responses to immunotherapy (Hales et al. 2010), were used in the current trial. Therefore, in subsequent studies on MGN1703, immune-related response criteria may be more appropriate.

Data from several randomised trials investigating maintenance treatment for metastatic CRC with agents such as bevacizumab have recently been reported (Díaz-Rubio et al. 2012; Tournigand et al. 2012; Koeberle et al. 2013; Koopman et al. 2014). Interpretation of these data is complex, as all the trials differ in design and in how the maintenance or de-escalation phase is managed. Therefore, many questions remain regarding whether, and in what form, maintenance treatment should be used in this setting. Comparison of PFS data from the current trial and other trials of maintenance therapy is made difficult by the different assessment schedules employed. In the study reported here, the first assessment was performed at month 3 to allow patients to receive adequate immunomodulatory therapy; this is reflected in the steep decay of the upper portions of the Kaplan–Meier curves. With regard to other immunomodulators, the current data appear to be consistent with observations from some trials showing no effect in terms of median PFS but lasting benefit in some patients (Chung et al. 2010; Kirkwood et al. 2010). It may be of interest to observe, as with other immunomodulators, whether mature OS results for MGN1703 point to a broader activity than seen on the shorter-term PFS endpoint.

Structural and preclinical studies have shown that MGN1703 is a potent TLR9 agonist with limited capacity for interactions with molecules outside its target structure (Schmidt et al. 2006b, 2008; Kapp et al., submitted). In addition to this specificity, the relevant cellular activation profile of MGN1703 is distinct to that of other TLR9 agonists, such as PF-3512676, which failed to show benefit compared with chemotherapy alone in two phase III trials in NSCLC (Hirsh et al. 2011; Manegold et al. 2012). Additional immunotherapy approaches have been investigated in patients with tumour types other than CRC. These include agents that target specific immune regulatory checkpoints and increase the endogenous antitumour immune response. For example, ipilimumab which binds to CTLA-4, has been shown to result in 2-year survival rates of 23.5 % in patients with metastatic melanoma (Hodi et al. 2010). The majority of adverse events with ipilimumab are consistent with the proposed mechanism of action and can be severe, and long-lasting (Hodi et al. 2010). Other immune checkpoint inhibitors have been developed to target the programmed cell death 1 receptor and prevent T-cell inactivation, with studies investigating their potential in melanoma and NSCLC (Pardoll and Drake 2012). The combination of MGN1703 with a checkpoint inhibitor could offer a potentially synergistic immunotherapeutic approach in this setting.

Safety data for MGN1703 were in accordance with those from the phase I trial (Weihrauch et al., submitted). MGN1703 was generally well tolerated, with most drug-related adverse events being mild or moderate. Common adverse events included injection-site reactions, fever and fatigue. Good tolerance of MGN1703 was confirmed by the lack of treatment interruption despite therapy for up to 30 months in patients experiencing prolonged response. In general, the tolerability profile of MGN1703 appears similar to that of therapeutic vaccines such as sipuleucel-T, rather than the checkpoint inhibitors (Frohlich 2012).

Overall, the results from this trial are encouraging and suggest a potential role for TLR9-targeted immunoactivation with MGN1703 in maintenance therapy of metastatic CRC after an effective first-line regimen inducing a tumour response. It is important, however, that the current results are not over-interpreted because of the very limited sample size due to the early study termination. A randomised, phase III trial of MGN1703 in the first-line/maintenance setting in patients with metastatic CRC is planned.

## Electronic supplementary material

Below is the link to the electronic supplementary material.

Supplementary material 1 (PDF 277 kb)
