# Supplementary material for: Maintenance treatment with the immunomodulator MGN1703, a Toll-like receptor 9 (TLR9) agonist, in patients with metastatic colorectal carcinoma and disease control after chemotherapy: a randomised, double-blind, placebo-controlled trial
Source: J Cancer Res Clin Oncol. 2014 May 10;140(9):1615–24. doi: 10.1007/s00432-014-1682-7 (PMC4131138; doi:10.1007/s00432-014-1682-7)
Supplement: Supplementary file 1 — Supplementary material 1 (PDF 277 kb) [file 432_2014_1682_MOESM1_ESM.pdf]

Web extra material (Appendix)

Maintenance treatment with the immunomodulator MGN1703, a toll-like receptor 9 (TLR9) agonist, in patients with metastatic colorectal carcinoma and disease control after chemotherapy: a randomised, double-blind, placebo-controlled trial

Journal of Cancer Research and Clinical Oncology

Hans-Joachim Schmoll, Burghardt Wittig, Dirk Arnold, Jorge Riera-Knorrenschild, Dieter Nitsche, Hendrik Kroening, Frank Mayer, Johannes Andel, Reinhard Ziebermayr, Werner Scheithauer

Corresponding author:

Hans-Joachim Schmoll

Department of Internal Medicine IV, Oncology/Hematology, Martin Luther University Halle-Wittenberg, Ernst-Grube-Str. 40, DE-06120 Halle, Germany

Email: hans-joachim.schmoll@uk-halle.de

Web extra material (Appendix): overall survival

Fig. S1 OS by treatment group from (a) randomisation and (b) the start of induction therapy

Data are immature (>50% of patients are censored)

CI confidence interval; *HR* hazard ratio; *mOS* median overall survival; *NR* not reached; *OS* overall survival

a

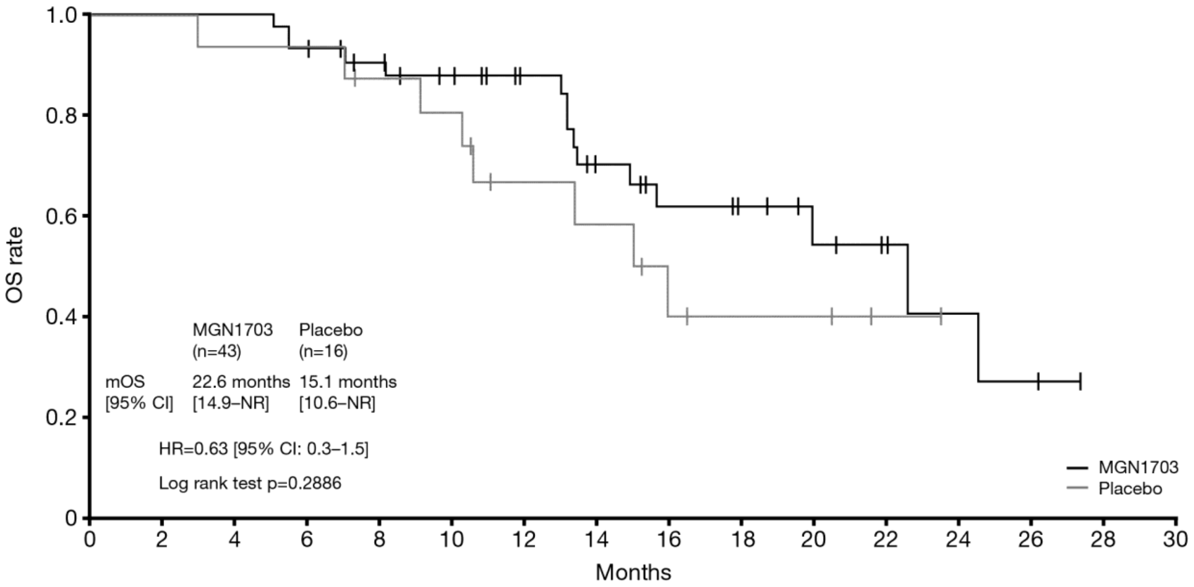

b

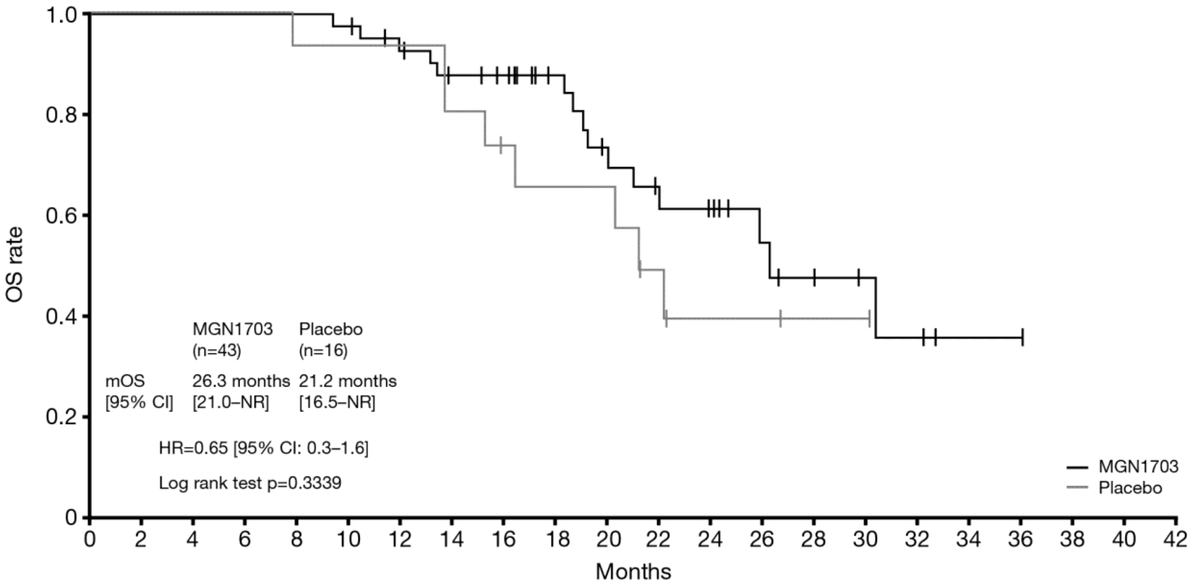

Data are immature (>50% of patients are censored)

Web extra material (Appendix): protocol-defined exploratory analyses of baseline patient characteristics predictive for progression-free survival

Methodology

Baseline characteristics evaluated included age, gender, Eastern Cooperative Oncology Group performance status, resection of primary tumour, *KRAS* mutation status, type and duration of first-line chemotherapy, change in tumour size and best response (Response Evaluation Criteria in Solid Tumors) following first-line chemotherapy, and levels of carcinoembryonic antigen, alkaline phosphatase, lactate dehydrogenase, albumin, serum glutamic-oxaloacetic transaminase, thrombocytes and lymphocytes. Significant baseline prognostic factors (level of significance set at 0.10) were selected and entered a multivariate Cox proportional hazards model.

Results

Fig. S2 Exploratory analysis of factors predictive of PFS

CEA carcinoembryonic antigen; *CI* confidence interval; *HR* hazard ratio; *PFS* progression-free survival; *SD* stable disease

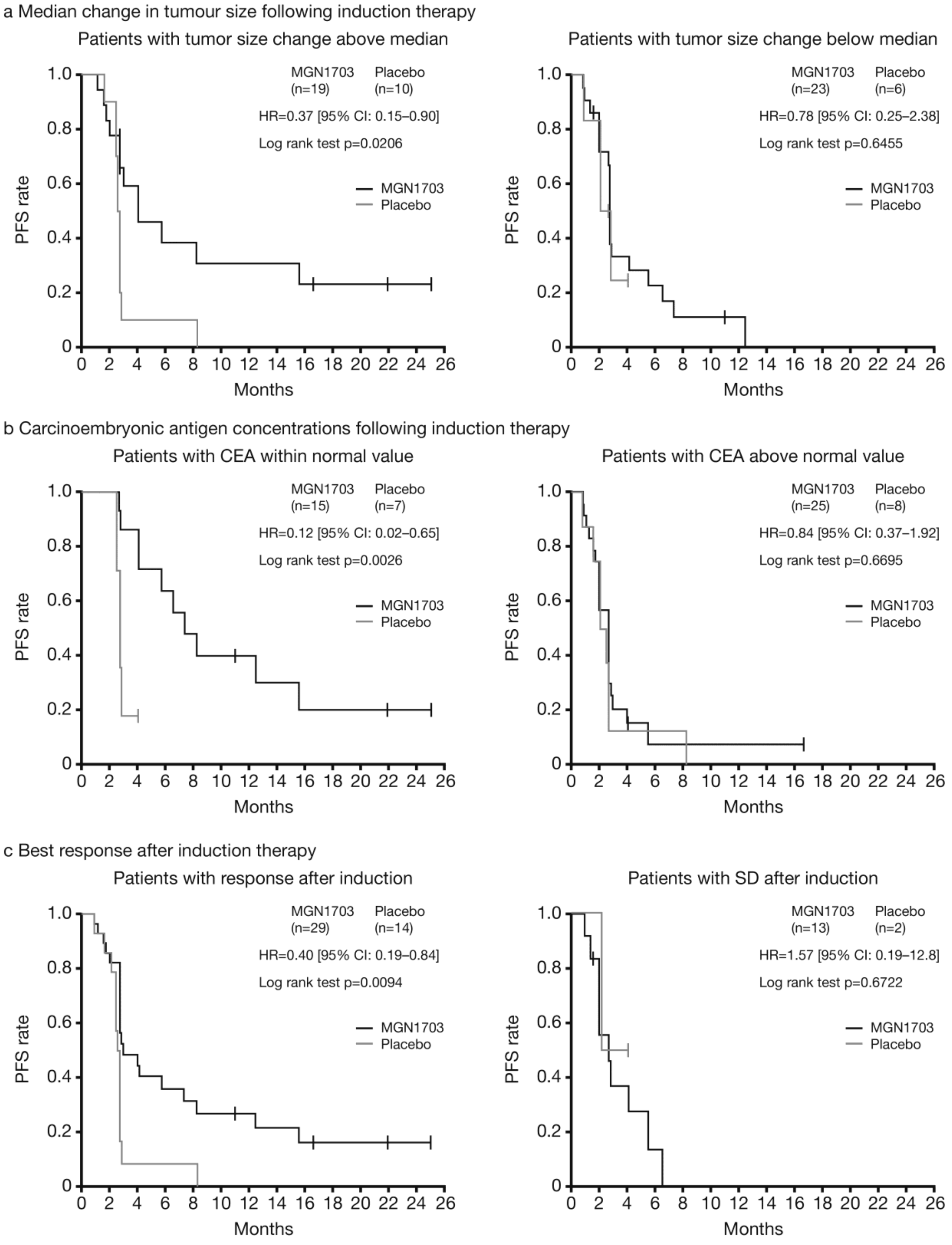
a Median change in tumour size following induction therapy
Patients with tumor size change above median
MGN1703 (n=19) Placebo (n=10)
HR=0.37 [95% CI: 0.15–0.90]
Log rank test p=0.0206
Patients with tumor size change below median
MGN1703 (n=23) Placebo (n=6)
HR=0.78 [95% CI: 0.25–2.38]
Log rank test p=0.6455
b Carcinoembryonic antigen concentrations following induction therapy
Patients with CEA within normal value
MGN1703 (n=15) Placebo (n=7)
HR=0.12 [95% CI: 0.02–0.65]
Log rank test p=0.0026
Patients with CEA above normal value
MGN1703 (n=25) Placebo (n=8)
HR=0.84 [95% CI: 0.37–1.92]
Log rank test p=0.6695
c Best response after induction therapy
Patients with response after induction
MGN1703 (n=29) Placebo (n=14)
HR=0.40 [95% CI: 0.19–0.84]
Log rank test p=0.0094
Patients with SD after induction
MGN1703 (n=13) Placebo (n=2)
HR=1.57 [95% CI: 0.19–12.8]
Log rank test p=0.6722
MGN1703
Placebo
PFS rate
Months

Web extra material (Appendix): immune system activation

Several cell populations (monocytes, B-lymphocytes, T-lymphocytes, NKT cells, NK cells, plasmacytoid dendritic cells and myeloid dendritic cells) were explored to evaluate the immunologic response to MGN1703. Monocytes and plasmacytoid dendritic cells were strongly activated, NK cells were activated and myeloid dendritic cells were not activated (Fig S3). NKT cells were activated, while T cells were slightly activated and B cells were not activated (figures not shown).

Fig. S3 Evidence for activation of the immune system by MGN1703 is shown by a treatment-induced increase in activated pDC in the total pDC population and the proportion of activated monocytes in the total monocyte population.

mDC myeloid dendritic cells*; MFI* mean fluorescence intensity value; *NK* natural killer cell; *pDC* plasmacytoid dendritic cell

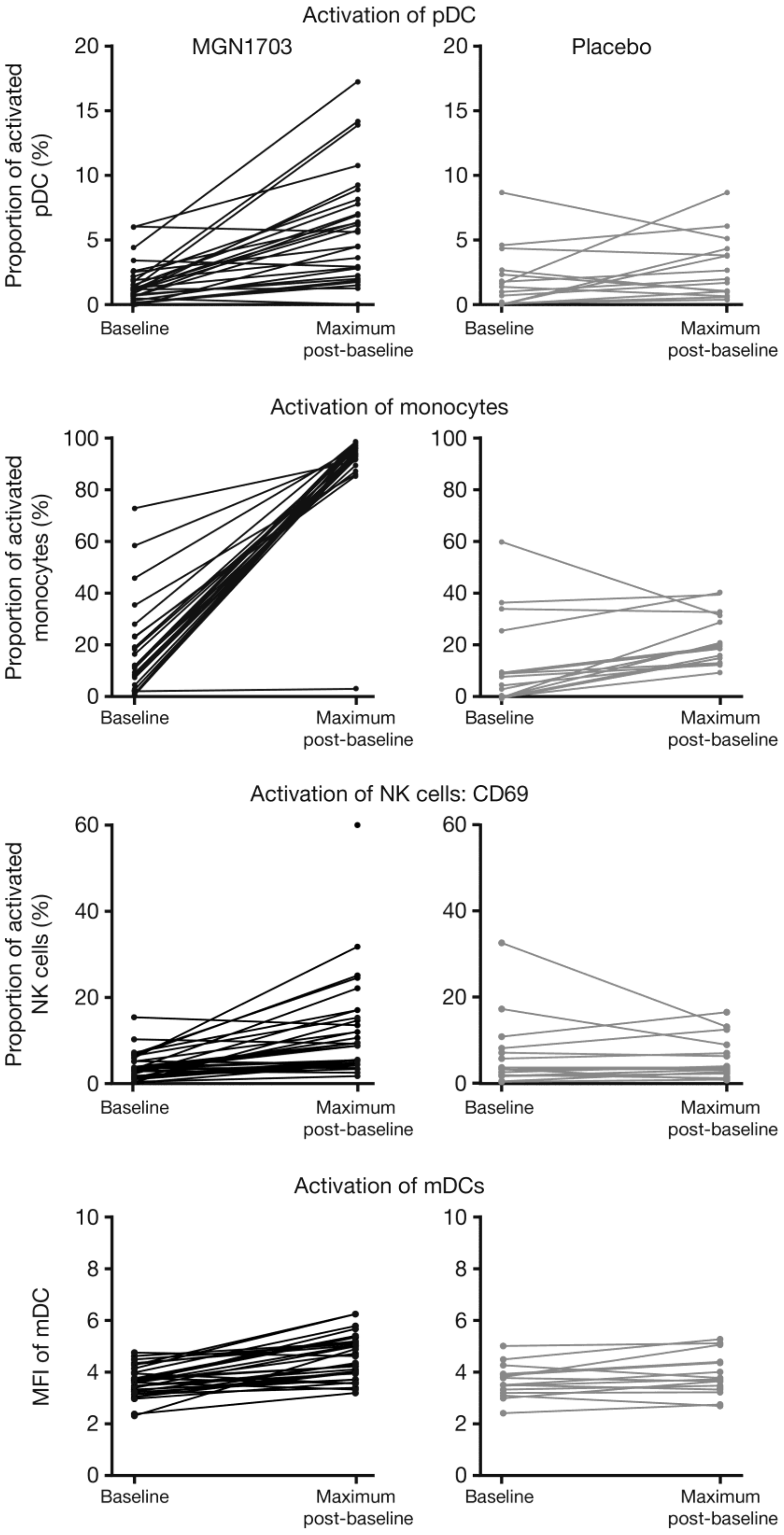
Activation of pDC
MGN1703
Placebo
Proportion of activated pDC (%)
0
5
10
15
20
Baseline
Maximum post-baseline
Activation of monocytes
Proportion of activated monocytes (%)
0
20
40
60
80
100
Activation of NK cells: CD69
Proportion of activated NK cells (%)
0
20
40
60
Activation of mDCs
MFI of mDC
0
2
4
6
8
10
